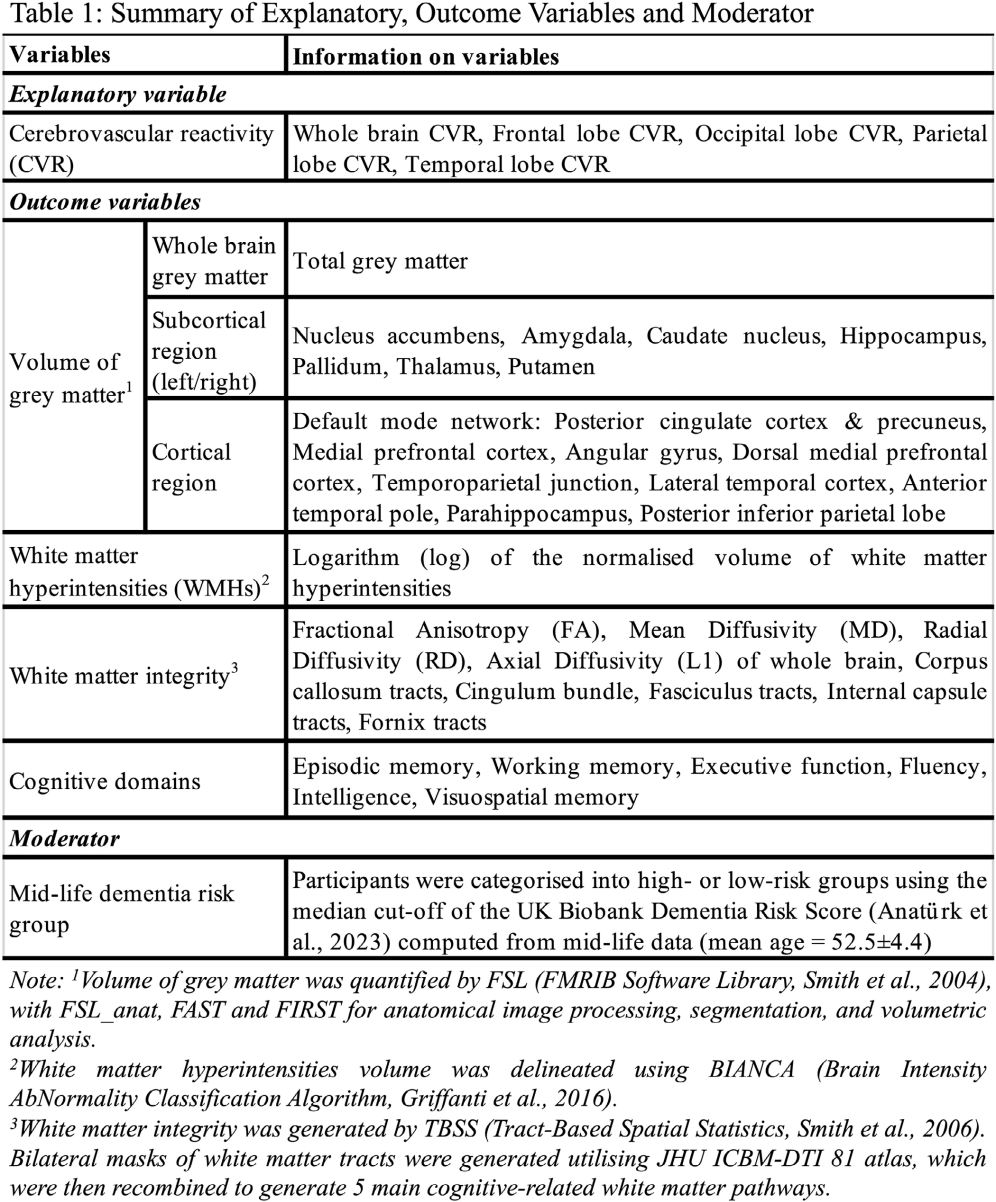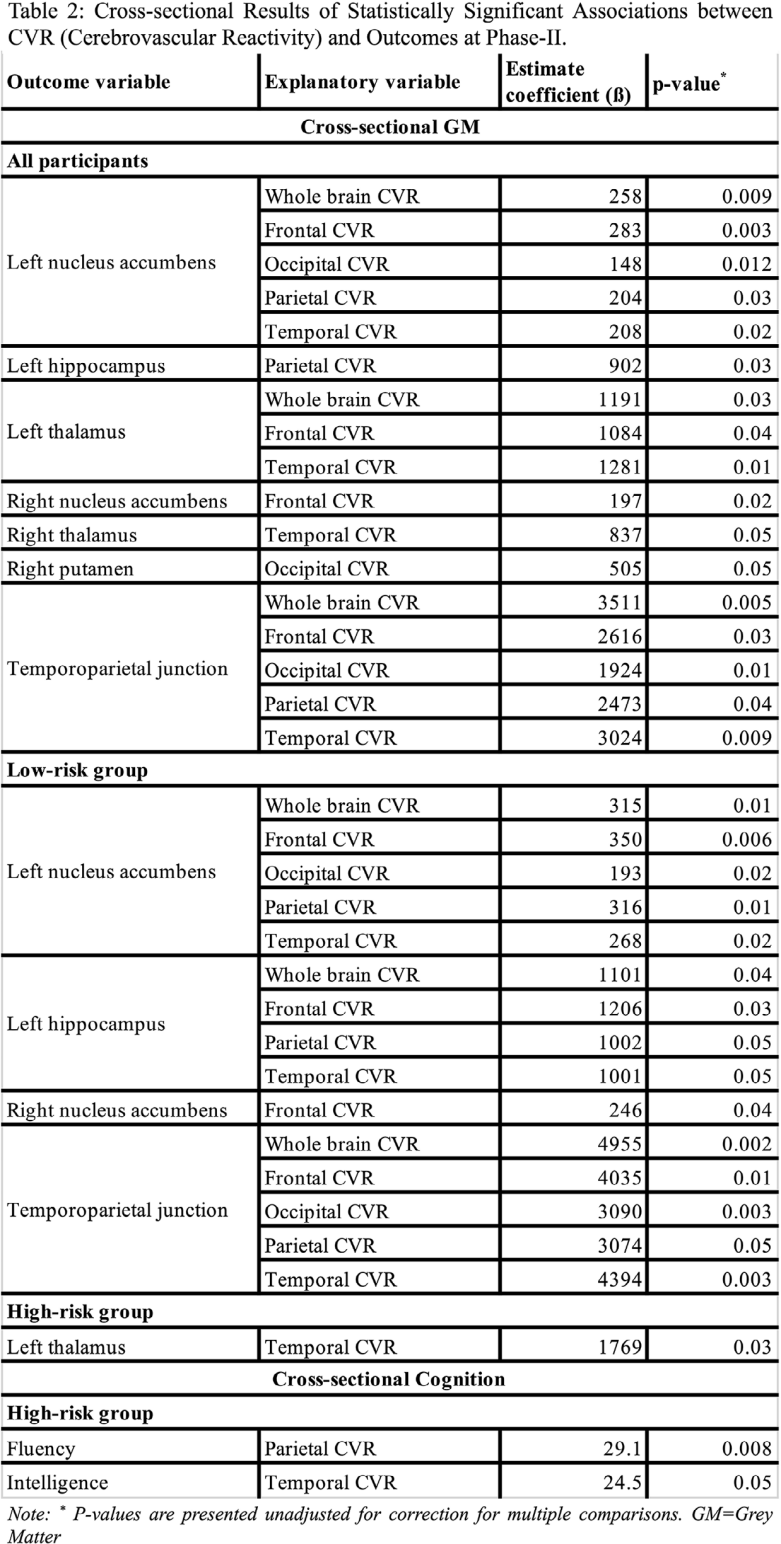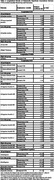# Associations between Later‐life Cerebrovascular Reactivity and Brain Structure and Cognitive Decline: A Longitudinal Study in an Aging Cohort with Varying Dementia Risks

**DOI:** 10.1002/alz.092261

**Published:** 2025-01-09

**Authors:** Congxiyu Wang, Raihaan Patel, Lucy Jobbins, Graham Reid, Georgina Hobden, Clare E Mackay, Klaus P Ebmeier, Mika Kivimäki, Archana Singh‐Manoux, Daniel Bulte, Sana Suri

**Affiliations:** ^1^ Department of Psychiatry, University of Oxford, Oxford UK; ^2^ Wellcome Centre for Integrative Neuroimaging, University of Oxford, Oxford UK; ^3^ Nuffield Department of Clinical Neurosciences, University of Oxford, Oxford UK; ^4^ Department of Experimental Psychology, University of Oxford, Oxford UK; ^5^ University of Helsinki, Helsinki Finland; ^6^ UCL Brain Sciences, University College London, London UK; ^7^ Universite de Paris, INSERM, Paris France; ^8^ Department of Engineering, University of Oxford, Oxford UK

## Abstract

**Background:**

Cerebrovascular reactivity (CVR) is implicated in the progression of dementia, though the underlying mechanisms is not understood. This study examines the relationships between CVR and brain structure and cognitive decline, moderated by mid‐life dementia risk.

**Method:**

163 participants from the Whitehall‐II cohort underwent neuropsychological testing and MRI, including T1‐weighted, FLAIR, and DTI sequences, at two phases (Phase‐I: mean age=68.2±4.4; Phase‐II: mean age=76.9±4.5). CVR was quantified via BOLD response to 5% CO_2_ only at Phase‐II. Linear regression tested the Phase‐II and Phase‐I to Phase‐II associations between CVR and brain and cognitive outcomes (Table 1), alongside its interaction with dementia risks. Post‐hoc analysis clarified the extent of these associations among different risk groups.

**Result:**

Tables 2 and 3 list significant cross‐sectional and longitudinal results, respectively. At Phase‐II, global CVR was positively associated with volume of left nucleus accumbens, and temporoparietal junction (p<0.03). Parietal CVR was positively associated with left hippocampus volume (p=0.03). These associations were more pronounced in the low‐risk group. Temporal CVR was related to thalamus volume (p<0.05) across all participants, with associations of the right thalamus exclusive to the high‐risk group (p=0.03). Longitudinally, lower global and regional CVR at Phase‐II was linked to greater reduction in temporoparietal junction volume (p<0.04). In high‐risk individuals, lower frontal, parietal, or global CVR was linked to larger volume declines in total grey matter or right thalamus respectively (p<0.05). Across all participants, lower parietal CVR at follow‐up was linked to greater FA reductions and RD increases between examinations in the corpus callosum (p=0.02) and to greater declines in FA and increases in MD, RD, and L1 in the cingulum bundle (p<0.04), with these effects being more pronounced in the low‐risk group. At Phase‐II, lower parietal and temporal CVR was associated with worse fluency and intelligence, respectively, in high‐risk individuals (p<0.05). Lower frontal CVR was linked to more executive function decline in the low‐risk group over‐time (p=0.03).

**Conclusion:**

This study highlights the differential impacts of global and regional CVR on brain structure and cognitive changes dependent on mid‐life dementia risks, which provides evidence for CVR as a potential biomarker for dementia and age‐related cognitive change.